# Engineering of self-rectifying filamentary resistive switching in LiNbO_3_ single crystalline thin film via strain doping

**DOI:** 10.1038/s41598-019-55628-3

**Published:** 2019-12-13

**Authors:** Tiangui You, Kai Huang, Xiaomeng Zhao, Ailun Yi, Chen Chen, Wei Ren, Tingting Jin, Jiajie Lin, Yao Shuai, Wenbo Luo, Min Zhou, Wenjie Yu, Xin Ou

**Affiliations:** 1 State Key Laboratory of Functional Material for Informatics, Shanghai Institute of Microsystem and Information Technology, Chinese Academy of Sciences, Shanghai, 200050 P. R. China; 2 Department of Physics, Shanghai University, Shanghai, 200444 P. R. China; 3 State Key Laboratory of Electronic Thin Films and Integrated Devices, University of Electronic Science and Technology of China, Chengdu, 610054 P. R. China

**Keywords:** Electronic devices, Electronic and spintronic devices

## Abstract

The abilities to fabricate wafer scale single crystalline oxide thin films on metallic substrates and to locally engineer their resistive switching characteristics not only contribute to the fundamental investigations of the resistive switching mechanism but also promote the practical applications of resistive switching devices. Here, wafer scale LiNbO_3_ (LNO) single crystalline thin films are fabricated on Pt/SiO_2_/LNO substrates by ion slicing with wafer bonding. The lattice strain of the LNO single crystalline thin films can be tuned by He implantation as indicated by XRD measurements. After He implantation, the LNO single crystalline thin films show self-rectifying filamentary resistive switching behaviors, which is interpreted by a model that the local conductive filaments only connect/disconnect with the bottom interface while the top interface maintains the Schottky contact. Thanks to the homogeneous distribution of defects in single crystalline thin films, highly reproducible and uniform self-rectifying resistive switching with large on/off ratio over four order of magnitude was achieved. Multilevel resistive switching can be obtained by varying the compliance current or by using different magnitude of writing voltage.

## Introduction

Due to the advantages of high-speed switching, large scalability, low energy consumption and multi-bit memory, resistive switching devices, comprised of a semiconductor (or insulator) sandwiched with metallic electrodes, are considered as one of the leading candidates for the next generation nonvolatile memories known as resistive random access memories (ReRAM)^1,2^. Additionally, resistive switching devices are also recognized as memristors^3,4^, whose resistance states depend upon the external applied voltage history. Resistive switching devices (or memristors) have attracted great attention thanks to the potential applications in the reconfigurable logics^5–8^ and neuromorphic computing^9–11^ which are the alternatives to the conventional transistor-based computer to overcome the von Neumann bottleneck^8^. To date, the resistive switching characteristics have been observed in a wide variety of binary and ternary oxide materials, such as TiO_2_^2,3,12^, HfO_2_^10,13,14^, BiFeO_3_^15–17^, SrTiO_3_^18–20^, and so on. In order to develop resistive switching materials to applicable memristor cells and to advance to the ultimate scaling limits for future ReRAM, deep understanding of the underlying resistive switching mechanisms is indispensable. Thanks to recent intensive investigations, there is a general agreement that the defects in the oxide thin films play an important role for the resistive switching^21,22^, because the defect distribution, crystallinity, and nonstoichiometry of the oxides influence the device’s transport properties, i.e., defects at interfaces or lattice strain lead to local compositional gradients and ionic near order modulations^23,24^.

Due to the lattice mismatch, it is rather difficult to fabricate high quality single crystalline oxide thin films on the metallic substrates by the conventional thin film growth methods, e.g., reactive magnetron sputtering, pulse laser deposition and so on. Thus, most of the reported investigations focus on the resistive switching characteristics of the polycrystalline or amorphous oxide thin films^7,13,16,19,25–27^, in which there are different types of defects and the distribution of the defects, the grain size and grain boundaries are uncontrollable. Therefore, it is great important to exploit the fabrication of single crystalline oxide thin films on the metallic substrates and to engineer its resistive switching characteristics. Conventionally, the single crystalline oxide thin films can be epitaxially grown on the conductive oxide substrates, e.g., Nb-doped SrTiO_3_^28–31^. However, the option of the oxide thin films is limited and it is not appropriate for the large scale mass fabrication. Alternatively, ion slicing with wafer bonding, which can transfer many kinds of large scale single crystalline thin films onto different substrates, would be a more feasible approach to fabricate single crystalline oxide thin films on the metallic substrates^32–34^. Resistive switching has been reported in some single crystalline oxide thin films^28–31,34^, and tuning the oxygen partial pressure during the thin film deposition or post-annealing in different atmosphere was employed to create the oxygen vacancies (OVs) in the single crystalline oxide thin films and to further control the resistive switching characteristics. However, these solutions only allow a global tuning of the resistive switching. The ability to locally engineer the resistive switching is therefore nontrivial to realize, which is essential for the fabrication of highly integrated hybrid nano-devices. Alternatively, introducing a slight lattice constant modification by noble gas implantation has been proved to be an effective approach to locally tailor the physical properties in semiconductors or functional oxides, e.g., the electronic transport and ferroelectric switching in BiFeO_3_ thin films can be engineered via He implantation^35^. This approach was recognized as “strain doping”^36,37^. The conventional epitaxy-based strain tuning methods are constrained not only by the Poisson effect but also by the limited set of available substrate. Alternatively, the ion implantation-based strain doping allows the independent and continuous strain engineering along a single axis^36^. Particularly, He implantation possesses the advantage that the helium’s stopping power in the lattice comes almost entirely from non-nuclear interactions, which minimizes damage to the film structure^38^. Moreover, helium’s nobility assures that no extra electron or holes are doped into the films as with hydrogen doping^39^.

In this work, the ion slicing with wafer bonding was employed to fabricate a congruent wafer scale LiNbO_3_ (LNO) single crystalline thin film on Pt/SiO_2_/LNO substrate. The lattice strain in LNO single crystalline thin films can be engineered by strain doping via He implantation, and consequently highly reproducible and uniform resistive switching with self-rectifying, large on/off ratio and multi-level switching was obtained.

## Methods

### Material preparation

**Figure 1:**
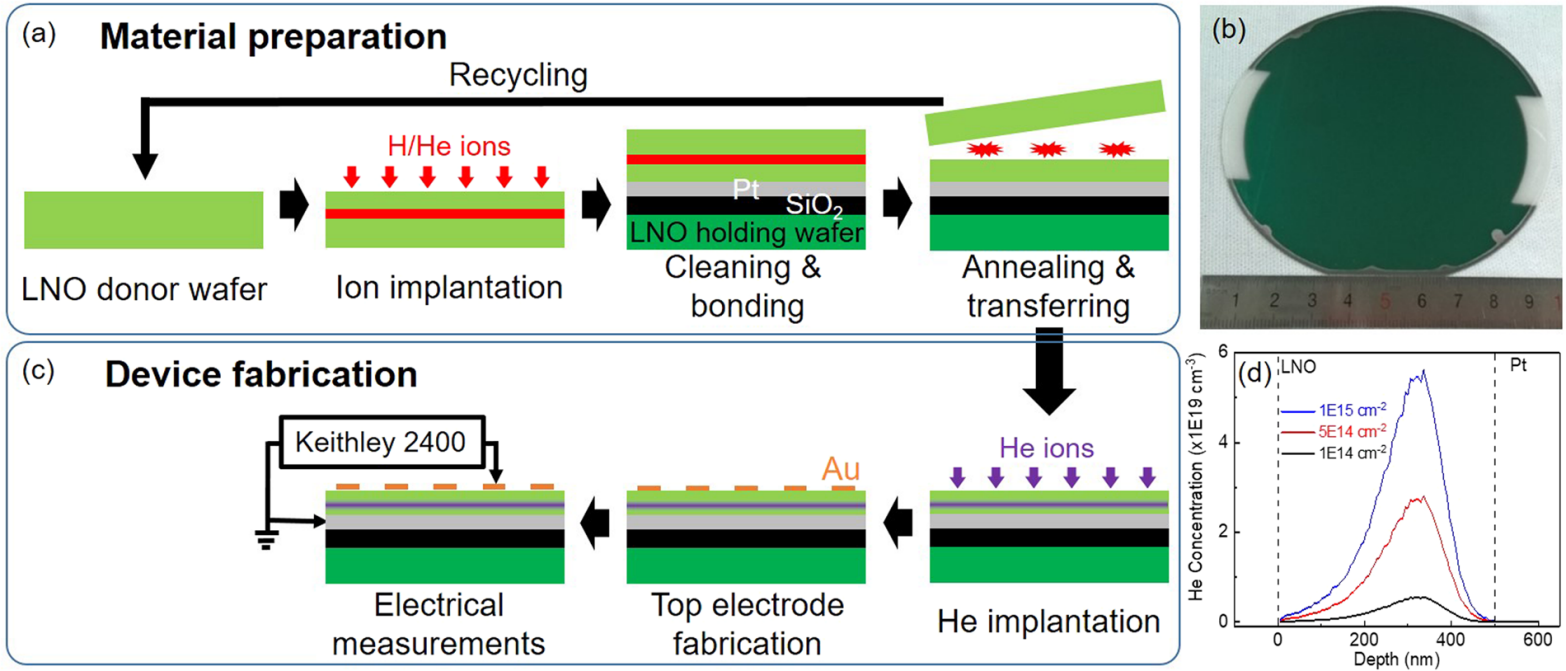
Schematic sketches of (**a**) materials preparation process and (**c**) device fabrication process, (**b**) the picture of as-prepared 4 inch LNO/Pt/SiO_2_/LNO sample, and (**d**) the simulation result of He distribution in the LNO thin films.

Wafer scale LNO single crystalline thin film on Pt/SiO_2_/LNO substrate was prepared by ion slicing with wafer bonding as shown in Fig. 1(a). H implantation was carried out on a 4-inch commercialized bulk LNO using a Nissin EXCEED 2300RD ion implanter. During the implantation, the wafer was tilted by 7° from normal to minimize the ion channeling effect. After a standard RCA cleaning process, the surfaces of both implanted LNO wafer and Pt/SiO_2_/LNO substrate were activated by N_2_ plasma, and then the implanted LNO wafer and Pt/SiO_2_/LNO substrate were directly bonded at room temperature with an EVG 301 wafer bonding system. After the bonding process, the bonding couple was annealed at 150 °C with N_2_ atmosphere for 2 hours. After that, the implanted LNO wafer was sliced at the target depth. A LNO single crystalline thin film was transferred to Pt/SiO_2_/LNO substrate and the remained LNO wafer can be recycled. Figure 1(b) shows the picture of the as-prepared 4 inch LNO/Pt/SiO_2_/LNO sample. There are two fringes on the left and right sides of the as-prepared 4 inch LNO/Pt/SiO_2_/LNO sample due to shadow effect of the chucks during the deposition of SiO_2_ and Pt by electron beam evaporation.

### Resistive switching device fabrication

As schematically shown in Fig. 1(c), a z-cut LNO (500 nm)/Pt (100 nm)/SiO_2_ (1 μm)/LNO (500 μm) was prepared, where z-cut LNO (500 nm) is the sliced and transferred single crystalline thin film, Pt serves as the bonding layer and bottom electrode, SiO_2_ serves as the insulate layer, and LNO (500 μm) serves as the holding substrate. The as-prepared sample of LNO/Pt/SiO_2_/LNO was cut into many small pieces with size of 5 mm × 5 mm, and then the He implantation on the LNO single crystalline thin films was carried out at room temperature with an ion energy of 60 keV. The He implantation fluences are 1E14 cm^−2^, 5E14 cm^−2^, and 1E15 cm^−2^, respectively. The He distribution in the LNO thin film was simulated by SRIM-2008^40^ as shown in Fig. 1(d). The implanted He ions are Gaussian distributed with the distribution peak closed to the Pt bottom electrode. Moreover, the He concentration increases with increasing He implantation fluence. After the He implantation, circular Au top contacts with a diameter of 200 μm and a thickness of 30 nm were prepared by DC magnetron sputtering at room temperature using a metal shadow mask. Thus, Au-LNO-Pt capacitor-like metal-insulator-metal (MIM) structures with different He implantation fluences were fabricated.

### Characterizations

The crystalline quality and microstructure of the LNO thin film were examined by X-ray diffraction (XRD) with a Philips X’Pert X-ray diffractometer and cross-sectional transmission electron microscopy (XTEM) with a JEOL 2100 F field-emission transmission electron microscopy. The electrical measurements were carried out by the two-probe method with a Keithley 2400 source meter at room temperature under dark condition. The DC bias voltage was applied on Au top electrode and Pt bottom electrode was grounded.

### First principle calculation

The Perdew-Burke-Ernzerhof (PBE) method as implemented in the Vienna Ab initio Simulation Package (VASP) was employed for the density functional theory (DFT) calculations. The electron exchange–correlation interaction was described by the generalized gradient approximation (GGA) plus the on-site Coulomb interaction (GGA + U) approach. The effective value of Hubbard U was chosen to be 4 eV on the Nb atoms, the kinetic energy cutoff was chosen as 500 eV for the plane wave basis set and the first Brillouin-zone integration was 3 × 3 × 3 Monkhorst-Pack grids. The convergence of electronic energy was set to 10^−6^ eV in the self-consistent calculations. During structural optimization, all atoms in the LNO were allowed to relax along any directions, and the interatomic forces were converged to less than 0.01 eV/Å.

## Results and Discussion

**Figure 2:**
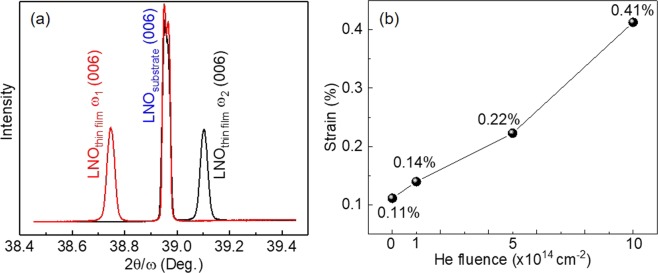
(**a**) The ɷ-2θ scans high-resolution XRD curves with opposite beam paths along the LNO (006) reflection of the virgin LNO thin film without He implantation. (**b**) The out-of-plane lattice strains of LNO thin films as a function of He implantation fluence.

XRD measurements were employed to study the crystalline quality and lattice strain of the LNO single crystalline thin films. It is noted that there is no lattice matching relationship between the transferred LNO thin films and the LNO holding substrate prepared by ion slicing with wafer bonding, which is different from the case of the traditional thin film growth. Thus, the accurate XRD peak position of the transferred thin film cannot be identified by that of the substrate. Alternatively, the Bond method was used to determine the absolute lattice parameters of the transferred thin films, with which two XRD measurements were carried out from the same set of planes but with opposite beam paths^41,42^. As shown in Fig. S1(a) in supporting information, the absolute XRD peak position (θ) can be calculated by θ = (ω_1_ + ω_2_)/2, where ω_1_ and ω_2_ are the XRD peak positions measured by the opposite beam paths, respectively. Figure 2(a) shows the ɷ-2θ scans with opposite beam paths along the LNO (006) reflection of the virgin LNO thin film without He implantation. The absolute LNO (006) peak position of the virgin LNO thin film is calculated to be θ_vir_ = 38.9248° which slightly deviates from that of LNO single crystal (θ_0_ = 38.9513°). This indicates that there is a little residual stress in the LNO thin film prepared by ion slicing with wafer bonding. The ɷ-2θ scans with opposite beam paths along the LNO (006) reflection of the He-implanted LNO thin films are shown in Fig. S1(b–d) in supporting information. With increasing He implantation fluence, the LNO thin film (006) peak shifts towards the smaller angles and splits into two distinct peaks, which suggests the out-of-plane lattice expansion and degradation of crystalline quality induced by He implantation. According to Bragg’s equation, 2*d*·sin *θ* = *nλ* (where *θ* is the diffraction angle, *λ* is the wavelength of the incident light (*λ* = 1.54 Å in this work), and *n* is the diffraction index) the lattice spacing (*d*) can be calculated. The lattice strain (*ε*) can be determined from the changes in lattice plane spacing using

$${\rm{\varepsilon }}=\frac{d-{d}_{0}}{{d}_{0}}$$

where *d*_0_ is the lattice spacing of the sample at the beginning of stable elastic deformation, i.e., the lattice spacing of the LNO single crystal in this work. The lattice expansion induced out-of-plane tensile lattice strains of LNO thin films with different He implantation fluences were calculated as listed in Fig. 2(b). The out-of-plane tensile lattice strain increases gradually with increasing He implantation fluence and it is 0.41% for the highest He implantation fluence of 1E15 cm^−2^.

**Figure 3:**
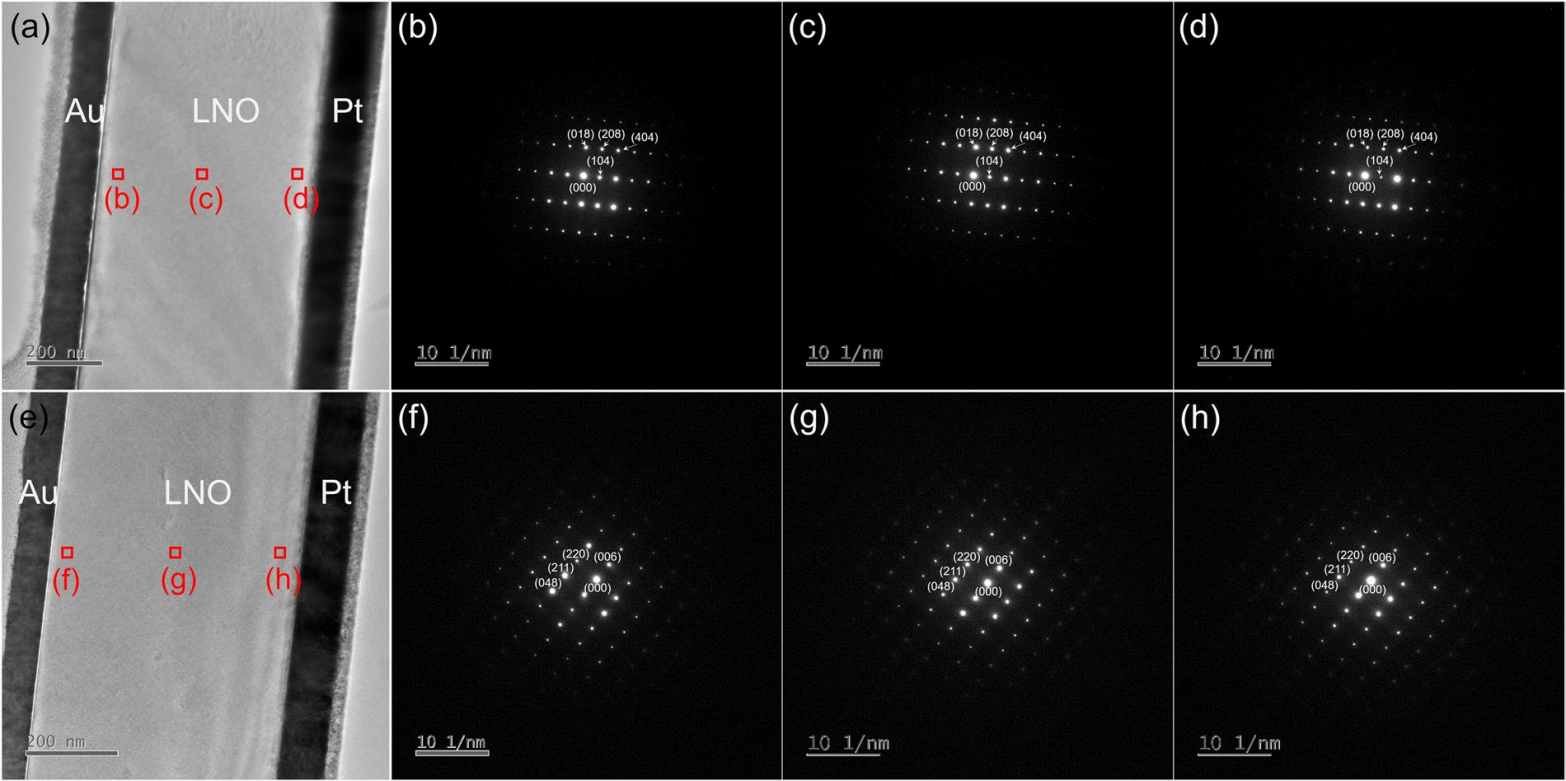
(**a**) Cross-sectional transmission electron microscopy (XTEM) image of the virgin sample, (**b**–**d**) the indexed selected area electron diffraction (SAED) patterns taken from the area of the top, middle and bottom of the LNO thin film in the virgin sample, respectively. (**e**) XTEM image of the sample with He implantation fluence of 1E14 cm^−2^, (**f**–**h**) the indexed SAED patterns taken from the area of the top, middle and bottom of the LNO thin film in the sample with He implantation fluence of 1E14 cm^−2^, respectively.

Even though the out-of-plane tensile lattice strain was induced in the LNO thin films by He implantation, the LNO thin films still maintained the the single crystalline quality as revealed by the XTEM images shown in Fig. 3. Figure 3(a) shows the XTEM image of the virgin sample, and Fig. 3(b–d) show the selected area electron diffraction (SAED) patterns taken from the area of the top, middle and bottom of the LNO thin film in the virgin sample, respectively. The XTEM image and the corresponding SAED patterns of the sample with He implantation fluence of 1E14 cm^−2^ are illustrated in Fig. 3(e–h), respectively. The thickness of the LNO thin films in both samples is about 500 nm as expected. All the SAED patterns show the regular single crystal diffraction patterns which can be indexed to the hexagonal phase of LiNbO_3_. Therefore, the single crystalline quality of the LNO thin films in both virgin sample and sample with He implantation fluence of 1E14 cm^−2^ is guaranteed.

**Figure 4:**
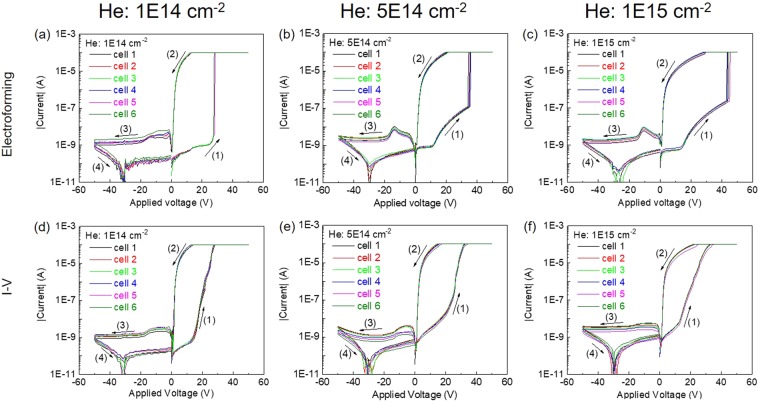
Electroforming process of 6 cells collected from the samples with He implantation fluence of (**a**) 1E14 cm^−2^, (**b**) 5E14 cm^−2^ and (**c**) 1E15 cm^−2^. I-V curves of 6 cells collected from the samples with He implantation fluence of (**d**) 1E14 cm^−2^, (**e**) 5E14 cm^−2^ and (**f**) 1E15 cm^-2^ after the electroforming process. The arrows indicate the bias voltage sweeping sequence.

The current-voltage (I-V) measurements were performed on the Au-LNO-Pt MIM structures with a Keithley 2400 source meter. The schematic sketch of the electrical measurement configuration is indicated in Fig. 1(c), in which the DC bias voltage was applied on Au top electrode and Pt bottom electrode was grounded. Unless noted otherwise, the DC bias voltage was swept in sequence of 0 V → + 50 V → −50 V → 0 V. To prevent the permanent breakdown of the MIM structures, a compliance current was set to 100 μA except where noted. As shown in Fig. S2(a) in supporting information, the virgin LNO thin film initially showed high resistance state (HRS), and an electroforming-like process occurred when the positive bias applied on the Au top electrode was ramped up to around +33 V for the first time. However, the virgin LNO thin film can not be reset to HRS and no significant current hysteresis was observed in the following I-V (current-voltage) cycle, which suggests that the Au-LNO-Pt MIM structures were permanent broken down. However, a typical electroforming process was observed on the He-implanted samples as shown in Fig. 4(a–c). Initially, all He-implanted samples exhibit high resistance state (HRS), and the electroforming occurs in the positive bias range with a sudden current increase which set the samples to low resistance state (LRS). For each He-implanted sample, more than 6 cells were electro-formed with the same recipe, and the electroforming process are highly consistent. It is widely believed that the sudden current increase in the electroforming process is associated with the formation of the conductive filaments in the dielectric material^21^. The electroforming voltage increases from 27.5 V to 43.5 V with the increasing He implantation fluence from 1E14 cm^−2^ to 1E15 cm^−2^ as indicated by the black curve in Fig. S2(b) in supporting information. However, before the sudden current increase there is an inflection point where the current increase starts to be enhanced for the samples with He implantation fluence of 5E14 cm^−2^ and 1E15 cm^−2^. Noted that the electroforming characteristics does not depend on the bias sweeping direction as indicated by Fig. S3 in supporting information. In Fig. S3, the bias voltage was swept in sequence of 0 V → −50 V → + 50 V → 0 V, while the I-V curves do not show significant different from that shown in Fig. 4(a–c).

After the electroforming process, each He-implanted sample exhibits uniform bipolar resistive switching behavior collected from 6 resistive switching cells as shown in Fig. 4(d–f). An obvious current hysteresis exists in the positive bias range with which the LRS is set by a positive writing bias and the HRS is reset by a negative writing bias. With the voltage sweeping from +50 V to −50 V, the I-V curves (branches (2) and (3) in Fig. 4(d–f)) exhibit a forward rectification characteristic, which suggests a Schottky contact formed at the Au/LNO top interface. The on/off ratio, which is defined as the current ratio of LRS and HRS at +5.0 V, decreases from ~53000 to ~3800 with increasing He implantation fluence suggested by the red curve in Fig. S2(b) in supporting information. By comparing the I-V curves of He-implanted samples shown in Fig. S2(c) in supporting information, it is obvious that larger current in HRS and in negative bias range is obtained with increasing He implantation fluence, which indicates that the conductivity of LNO thin films is increased by He implantation. However, the current in LRS shows the opposite pattern, which decreases with increasing implantation fluence. Thus, a large on/off ratio of ~53000 was achieved with low He implantation fluence of 1E14 cm^−2^.

**Figure 5:**
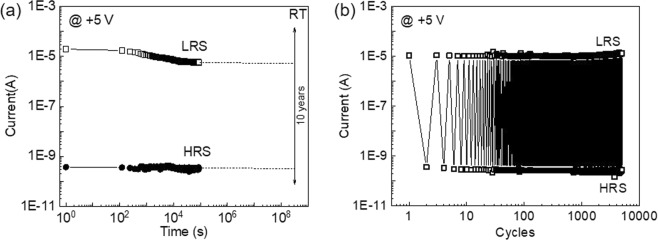
(**a**) Retention test results at room temperature (RT) for the sample with He implantation fluence of 1E14 cm^-2^. The extrapolated 10-year HRS/LRS retention time can be expressed by the dsashed line. (**b**) Endurance test result at RT for the sample with He implantation fluence of 1E14 cm^-2^.

To check the nonvolatility and reliability of the resistive switching observed in the He-implanted samples, the pulse retention and cycling endurance tests were performed. As shown in Fig. 5(a), the pulse retention tests were carried out by first setting/resetting the MIM structure to LRS/HRS with a writing bias of +50/−50 V and then detecting the resistance with a reading bias of +5 V every 2 min. It is clear that the HRS is relatively stable without significant variation during the testing period of 24 hours. However, the LRS initially shows an increasing resistance (decreasing current), but it becomes stable after around 6 hours. The on/off ratio of the sample with He implantation fluence of 1E14 cm^−2^ is still above four orders of magnitude after 24 hours. As indicated by the dashed lines in Fig. 5(a), the on/off ratio can be well-kept for more than 10 years at room temperature. The I-V characterization and pulse retention tests of the sample with He implantation fluence of 1E14 cm^−2^ were also carried out at an elevated temperature of 85 °C as shown in Fig. S4 in supporting information. The on/off ratio of the sample with He implantation fluence of 1E14 cm^−2^ at 85 °C is still above three orders of magnitude even after 24 hours and above two orders of magnitude after 10 years as indicated by the dashed line in Fig. S4(a) in supporting information, which suggests stable resistive switching characteristics. The endurance test was carried out at room temperature by repeating the set/read/reset/read process with short voltage pulses for more than 5000 cycles for the sample with He implantation fluence of 1E14 cm^−2^. A voltage pulse of +50 V/−50 V with a pulse width of 0.1 s was used as the set/reset bias, and the current values were obtained at a reading pulse of +5 V with a pulse width of 0.1 s in each switching cycle as shown in Fig. 5(b). The sample exhibits narrow distributions of both LRS and HRS, which suggests a repeatable and robust resistive switching behavior.

**Figure 6:**
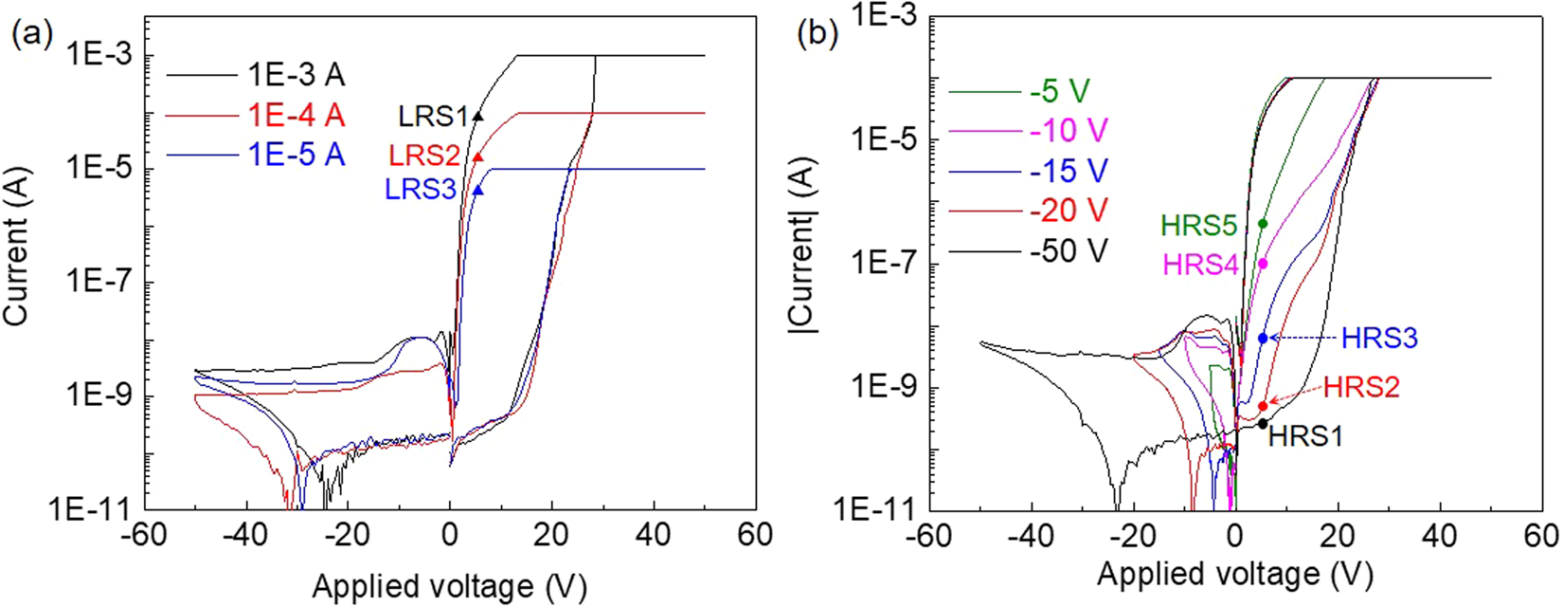
Multilevel resistive switching characteristics of the sample with He implantation fluence of 1E14 cm^-2^. (**a**) I-V curves with three different compliance currents (CC) reveal different LRS. (**b**) I-V curves with different negative maximum voltages (V_-max_) reveal different HRS.

Since the high on/off ratio is giant and abrupt, multilevel resistive switching could be exploited for the sample with He implantation fluence of 1E14 cm^−2^. By variation of the complicane current we were able to demonstrate the multilevel resistive switching. Fig. 6(a) shows the I-V characteristics with different compliance current of 1E-3 A, 1E-4 A and 1E-5 A, in which the HRS does not vary significantly while distinguishably different LRS (LRS1, LRS2 and LRS3) can be found for different compliance current. The current of LRS increases with increasing compliance current (CC). However, the resistive switching was not stable with large CC. After several resisitive switching cycles, the resistive switching devices were permanent broken when the CC was increased up to 1E-3 A as shown in Fig. S5 in supporting information. On the other hand, multilevel resistive switching can be achieved by using different magnitude of writing bias as well. As shown in Fig. 6(b), different negative maximum voltages (V_-max_) but the same positive maximum voltage were used in the I-V measurements. The LRS is similar while significantly different HRS (HRS1, HRS2, HRS3, HRS4 and HRS5) can be obtained with different negative maximum voltages of −50 V, −20 V, −15 V, −10 V and −5 V. The current of HRS increases with decreasing V_-max_. It is worth noting that the resistive switching presented in this work requires large operation voltage. As the peeled off LNO thin films are thick, a large operation voltage is needed to achieve a sufficient electric field for the resistive switching. An operation voltage as large as 200 V was reported when the “thickness” of the resistive switching region was around 2.5 μm^43^. By using a smaller implantation energy in the ion slicing process or by using an additional film thinning process, the thin film thickness can be reduced which could lower the resistive switching operation voltage. A comparison of the resistive switching performance of LNO thin films reported in this work and some of the resistive switching devices was summarized in Table S1 in supporting information. It suggests that the threshold electric fields for the resistive switching of LNO thin films (E_set_/E_reset_) in this work are in the average level in Table S1. The on/off ratio and retention time of LNO devices are comparable with those of the other recent reported resistive switching devices. The endurance of LNO devices was measured up to 5000 cycles in this work, which requires to be investigated in detail for further practical application purpose. The switching speed and the reading power consumption for LRS/HRS of LNO devices are tentatively much larger than those of the other recent reported resistive switching devices, which need to be further optimized.

**Figure 7:**
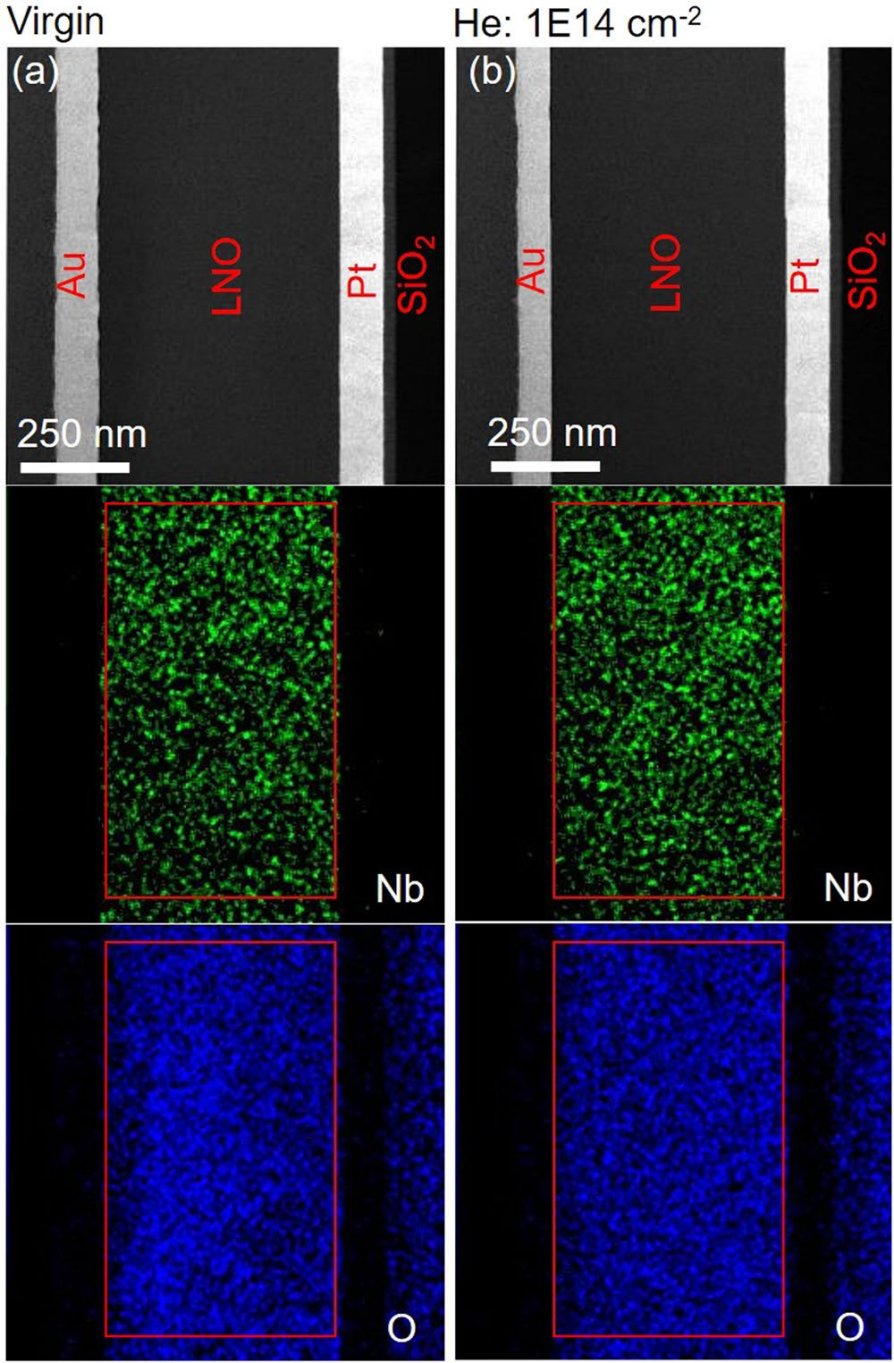
Scanning transmission electron microscopy-dark-field (STEM-DF) images and corresponding elemental maps (Nb and O) of the virgin sample (**a**) and the sample with He implantation fluence of 1E14 cm^-2^ (**b**).

**Figure 8:**
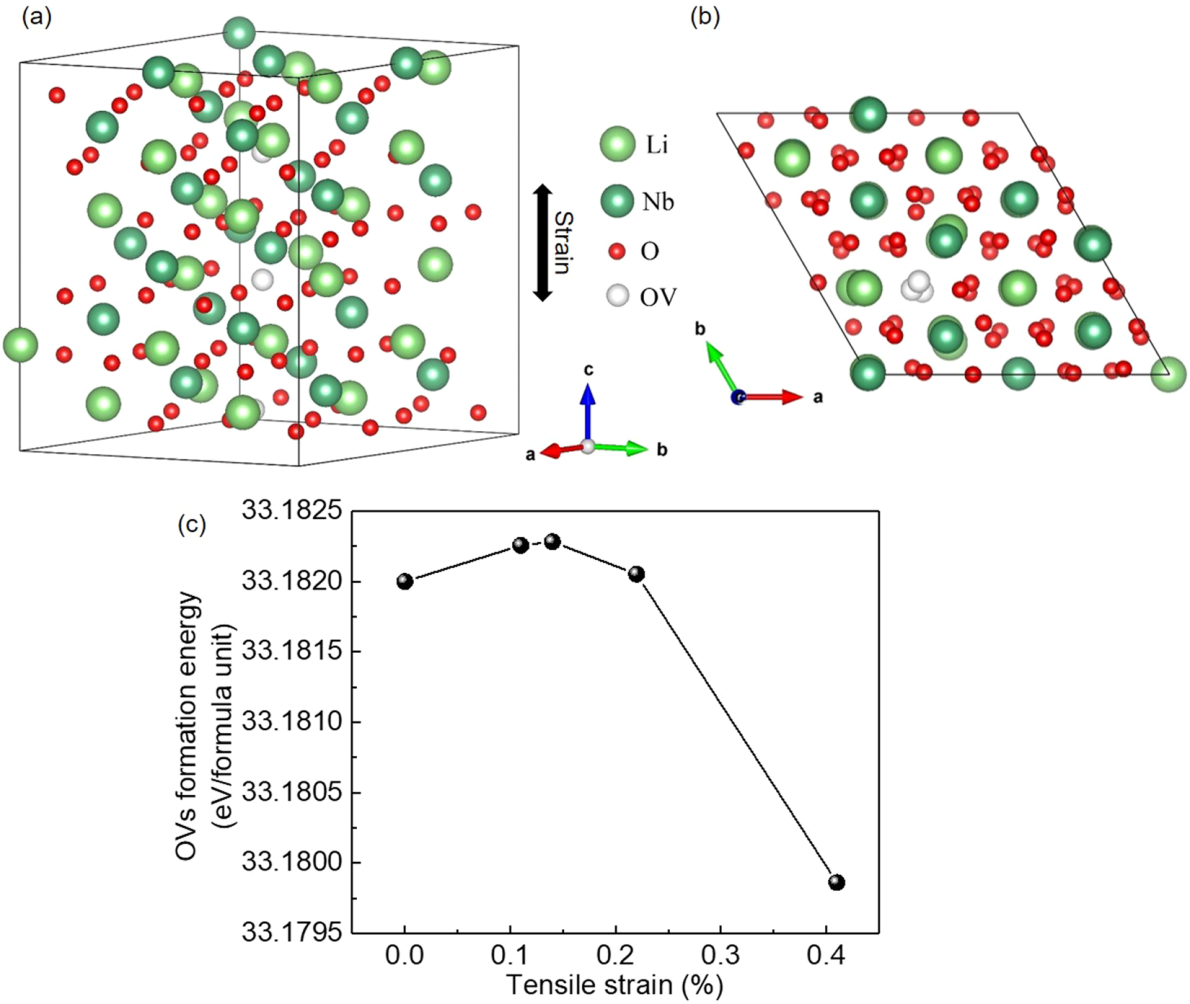
Lateral view (**a**) and top view (**b**) of the supercell structure diagram of LNO containing 3 OVs along the [001] direction. (**c**) Formation energy of ordered OVs under tensile strains up to 0.41%.

According to the above discussion, it is suggested that He implantation comes as an effective method to engineer the resistive switching in Au-LNO-Pt MIM structures. During the noble gas implantation, e.g., He implantation in this work, O atoms can be displaced in the oxides, e.g., LNO in this work, which creates stable oxygen vacancies (OVs) due to the low formation energy of OVs^44,45^. The existence of OVs in the LNO thin films was also confirmed by the energy dispersive spectroscopy (EDS) elemental mapping as shown in Fig. 7 and Table 1. Figure 7(a,b) show the dark-field scanning transmission electron microscopy (STEM-DF) and the corresponding EDS elemental mapping (Nb and O) for the virgin sample and the sample with He implantation fluence of 1E14 cm^−2^, respectively. There are 4 different layers identified in the STEM-DF images, i.e., Au, LNO, Pt, and SiO_2_. According to the EDS mapping, Nb was only observed in the LNO layer, while O appeared in the layers of LNO and SiO_2_. It is noted that the element of Li is not detectable by our TEM equipment. Table 1 lists the elemental composition (Atom%) of Nb and O in the regions within the LNO layer indicated by the red rectangular shown in the EDS mappings. For the virgin sample, the Nb and O content is 26.20% and 73.80%, respectively, and the atom ratio of O:Nb was calculated to be around 2.82, which is slightly below the stoichiometric ratio of 3 for LNO. This suggests the existence of the OVs in the LNO layer. While the sample with He implantation fluence of 1E14 cm^−2^ possesses a lower O:Nb atom ratio of 2.70 with the Nb and O content of 27.02% and 72.98%. This indicates that the OVs were introduced in the LNO thin films by He implantation. Additionally, the strain induced by the He implantation can further reduce the formation energy of OVs in the oxides^46–48^. The OVs act as mobile donors in the oxides and play an important role for the resistive switching^16,49–52^. The strain induced by the He-implantation can be employed to tune the formation energy of ordered OVs and to further engineer the distribution of OVs^53^. The first principle calculations were employed to identify the effect of the external strain on the formation energy of the ordered OVs in LNO. Figure 8(a,b) show a $2\sqrt{2}\times 2\sqrt{2}\times 3$ supercell structure diagram of LNO containing 3 OVs along the [001] direction. The out-of-plane tensile lattice strains was applied in the z direction by rescaling the lattice parameters. The formation energy of ordered OVs, *E*_vf_, serving as a measure of the strain response of oxygen defect chemistry, was calculated for out-of-plane tensile lattice strains from 0% to 0.41% as estimated by XRD measurements. *E*_vf_ was calculated as^53^

$${E}_{vf}=E(LN{O}_{1-x})-E(LNO)+n{\mu }_{O}$$

where *E*(LNO_1-x_) is the total energy of a LNO supercell with the ordered OVs, *E*(LNO) is the total energy of a perfect LNO supercell, *μ*_*O*_ is the oxygen chemical potential in LNO, and *n* is the number of OVs. As shown in Fig. 8(c), the formation energy of ordered OVs are calculated as a function of the applied out-of-plane tensile lattice strains. The formation energy of ordered OVs first increases but then decreases with increasing out-of-plane tensile lattice strains, which reaches the maximum at the tensile strain of 0.14% with He implantation fluence of 1E14 cm^−2^. The ordered OVs can serve as the conductive filaments in the oxides, which contributes to the resistive switching. Therefore, He implantation can be employed to control the resistive switching in oxides via strain engineering.

**Table 1:** Elemental composition (Atom%) of the regions indicated by the red rectangular shown in Fig. 7.

Element	Virgin	He: 1E14 cm^-2^
Nb	26.20	27.02
O	73.80	72.98

**Figure 9:**
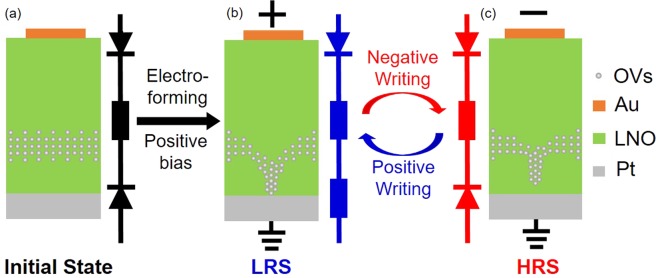
Schematics of the possible resistive switching mechanism. (**a**) Before the electroforming process, there is no filament formed in the LNO thin film (Initial State), and Schottky-like barriers are formed at both top and bottom interfaces. (**b**) In LRS, the filament is formed and connected to the Pt bottom electrode, and the bottom Schottky-like barrier is turned to a resistor. (**c**) In HRS, the filament is disconnected to the Pt bottom electrode, and the bottom Schottky-like barrier is recovered.

Both electroforming and rectifying behaviors appear in the resistive switching characteristics of Au-LNO-Pt MIM structures, which indicates the coexistence of the filamentary and interface resistive switching. The coexistence of the filamentary and interface resistive switching was also suggested by the I-V measrements of the samples with different cell diameters as shown in Fig. S6 in supporting information. The possible mechanism of the self-rectifying filamentary resistive switching observed in the Au-LNO-Pt MIM structures can be explained by the following model. Initially, the OVs were introduced in the LNO thin films by He implantation, and the distribution of OVs is in accordance with that of implanted He which indicates that the OVs gather below the middle of the LNO thin films as schematically shown in Fig. 9(a). The Schottky-like barriers can be formed at both top Au-LNO and bottom LNO-Pt interfaces, and the current is limited by the reversed Schottky-like barriers and the bulk resistance of LNO thin film before the electro-forming. With a positive forming bias, the positively charged OVs migrate towards the bottom LNO-Pt interface and form a conductive filament in the LNO thin film. The steep increase of current takes place when conductive filament reaches the Pt bottom electrode as shown in Fig. 9(b) and the Au-LNO-Pt MIM structure is set to LRS. The conductive filament does not penetrate throughout the whole LNO thin film. One end of the conductive filament touches the bottom Pt electrode, thus the bottom Schottky-like barrier is collapsed. While the other end of the conductive filament does not connect to the top Au electrode and the top Schottky-like barrier is remained. Therefore, the equivalent circuit of the Au-LNO-Pt MIM structure in LRS is a forward diode which shows a forward rectifying characteristic. The crystalline quality is degraded with large He implantation fluence as suggested by the XRD measurements, which will enlarge the energy barrier for the migration of OVs due to the anisotropic strain induced by the lattice deformation^54^. Therefore, the electro-forming voltage increases with high implantation fluence. However, the results of the first principle calculation suggest that the formation energy of ordered OVs decreases with increasing He implantation fluence over 1E14 cm^−2^. For the samples with He implantation fluence of 5E14 cm^−2^ and 1E15 cm^−2^, the conductive filaments might have been formed by the ordered OVs with a small voltage below the electroforming voltage but did not connect to the Pt bottom electrode. Therefore, the bulk resistance of the LNO thin film was lowered and an inflection point for the current increase was observed before the electro-forming as shown in Fig. 4(b,c). With a negative writing bias, the OVs are repelled from the bottom LNO-Pt interface and the conductive filament is disconnected with the Pt bottom electrode as shown in Fig. 9(c). The bottom Schottky-like barrier is rebuilt and the Au-LNO-Pt MIM structure is reset to HRS. In the following switching, the conductive filaments are connected/disconnected only with the Pt bottom electrode, while the Au-LNO top interface always maintains a Schottky-like barrier which functions as a “diode” in itself. Due to the enlarged migration barrier of OVs for the samples with high implantation fluence, the conductive filaments are not fully connected/disconnected with the Pt bottom electrode, which results in the smaller on/off ratio decreases for high He implantation fluence. The self-rectifying effect can effectively eliminate the sneak current in a crossbar architecture. Therefore, the selector usually used for the crossbar integration is not required, which will significantly improve the size of the crossbar array and simplify the fabrication process.

## Conclusions

In summary, wafer scale LNO single crystalline thin films have been prepared on Pt/SiO_2_/LNO substrates by ion slicing with wafer bonding. Strain doping with He implantation was employed to tune the lattice strain of the LNO single crystalline thin films, by which the self-rectifying filamentary resistive switching was achieved and engineered. The resistive switching mechanism was understood by a model that the local conductive filaments composed of OVs only connect/disconnect with the bottom electrode while the top interface maintains the Schottky contact. Thanks to the homogeneous distribution of defects, the single crystalline oxide thin films enable the highly reproducible and uniform self-rectifying resistive switching with large on/off ratio over four order of magnitude. The large on/off ratio allows the multilevel resistive switching by varying the compliance current or by using different magnitude of writing voltage. This work demonstrates a promising opportunity for the fabrication of wafer scale single crystalline oxide thin films on metallic substrates with controllable and uniform self-rectifying resistive switching, which might promote the industrialization development of resistive switching devices. By transferring different layers of single crystalline oxide thin films with patterned electrodes between the layers, these techniques are also adaptable for future large-scale data storage devices where memory cells are patterned in cross-point array and stacked in 3D structures.

## Supplementary information

Supporting Information
