# Supplementary material for: Engineering of self-rectifying filamentary resistive switching in LiNbO3 single crystalline thin film via strain doping
Source: Sci Rep. 2019 Dec 13;9:19134. doi: 10.1038/s41598-019-55628-3 (PMC6911103; doi:10.1038/s41598-019-55628-3)
Supplement: Supplementary file 1 — Supporting Information [file 41598_2019_55628_MOESM1_ESM.docx]

**Supporting Information**

Engineering of self-rectifying filamentary resistive switching in LiNbO_3_ single crystalline thin film via strain doping

Tiangui You,^1, †^ Kai Huang,^1, †^ Xiaomeng Zhao,^1^ Ailun Yi,^1^ Chen Chen,^2^ Wei Ren,^2^ Tingting Jin,^1^ Jiajie Lin,^1^ Yao Shuai,^3^ Wenbo Luo,^3^ Min Zhou,^1^ Wenjie Yu,^1^ and Xin Ou^1, *^

^1^ State Key Laboratory of Functional Material for Informatics, Shanghai Institute of Microsystem and Information Technology, Chinese Academy of Sciences, Shanghai 200050, P. R. China

^2^ Department of Physics, Shanghai University, Shanghai 200444, P. R. China

^3^ State Key Laboratory of Electronic Thin Films and Integrated Devices, University of Electronic Science and Technology of China, Chengdu 610054, P. R. China

* ouxin@mail.sim.ac.cn

^†^ These authors contributed equally to this work.


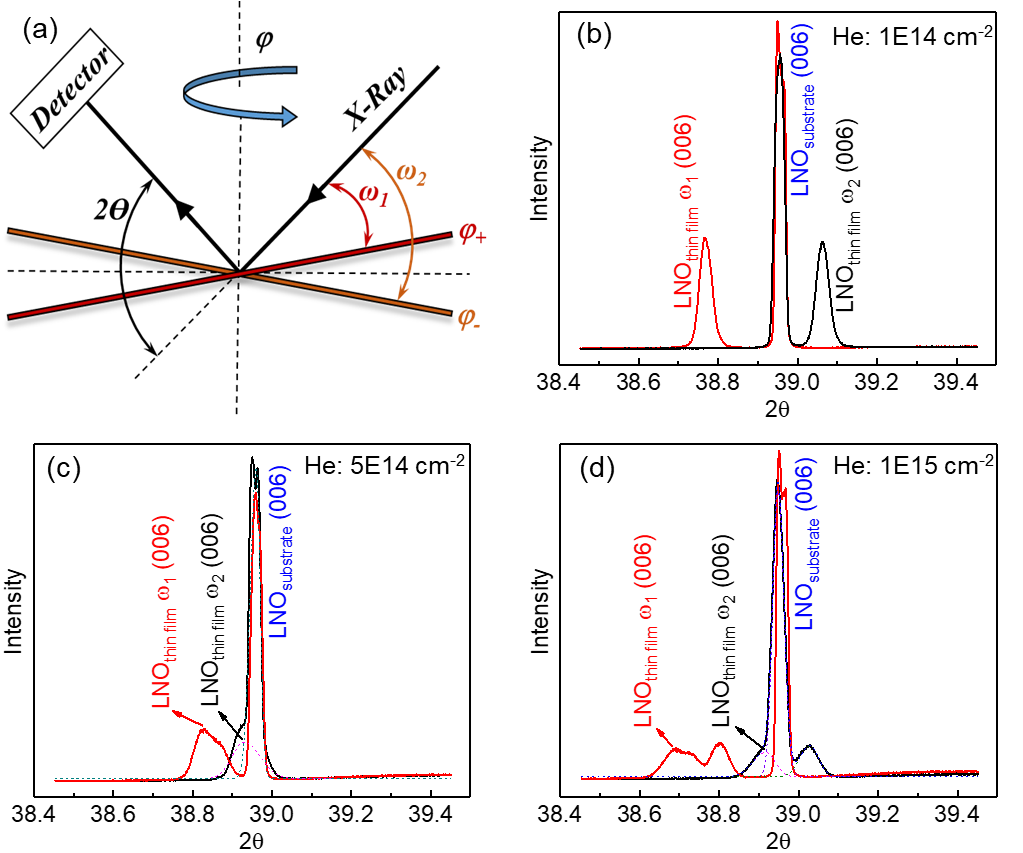


**Figure S1.** (a) Schematic sketch of the XRD measurement with Bond method. The ɷ-2θ scans with opposite beam paths along the LNO (006) reflection of the He-implanted LNO thin films with He implantation dose of (b) 1E14 cm^-2^, (c) 5E14 cm^-2^, and (d) 1E15 cm^-2^.

Bond method was used to identify the accurate XRD peak position of the transferred thin film. As shown Figure S1 (a), 2θ is the scattering angle between the incident and reflect light, ω denotes the angle between the sample surface and the direction of the incident light, φ quantifies the azimuthal orientation of the sample surface. The X-ray diffraction rocking curves on the LNO (006) plane were measured with the two different values of φ_+_ and φ_-_ (|φ_+_-φ_-_|=180 °), respectively. Thus two different values of ω_1_ and ω_2_ can be achieved in these two different positions. And the absolute XRD peak position (θ) can be calculated by θ=(ω_1_+ω_2_)/2. Figure S1 (b)-(d) show the ɷ-2θ scans with opposite beam paths along the LNO (006) reflection of the He-implanted LNO thin films. With increasing He implantation dose, the LNO thin film (006) peak shifts towards the smaller angles and splits into two distinct peaks, which suggests the out-of-plane lattice expansion and degradation of crystalline quality induced by He implantation.


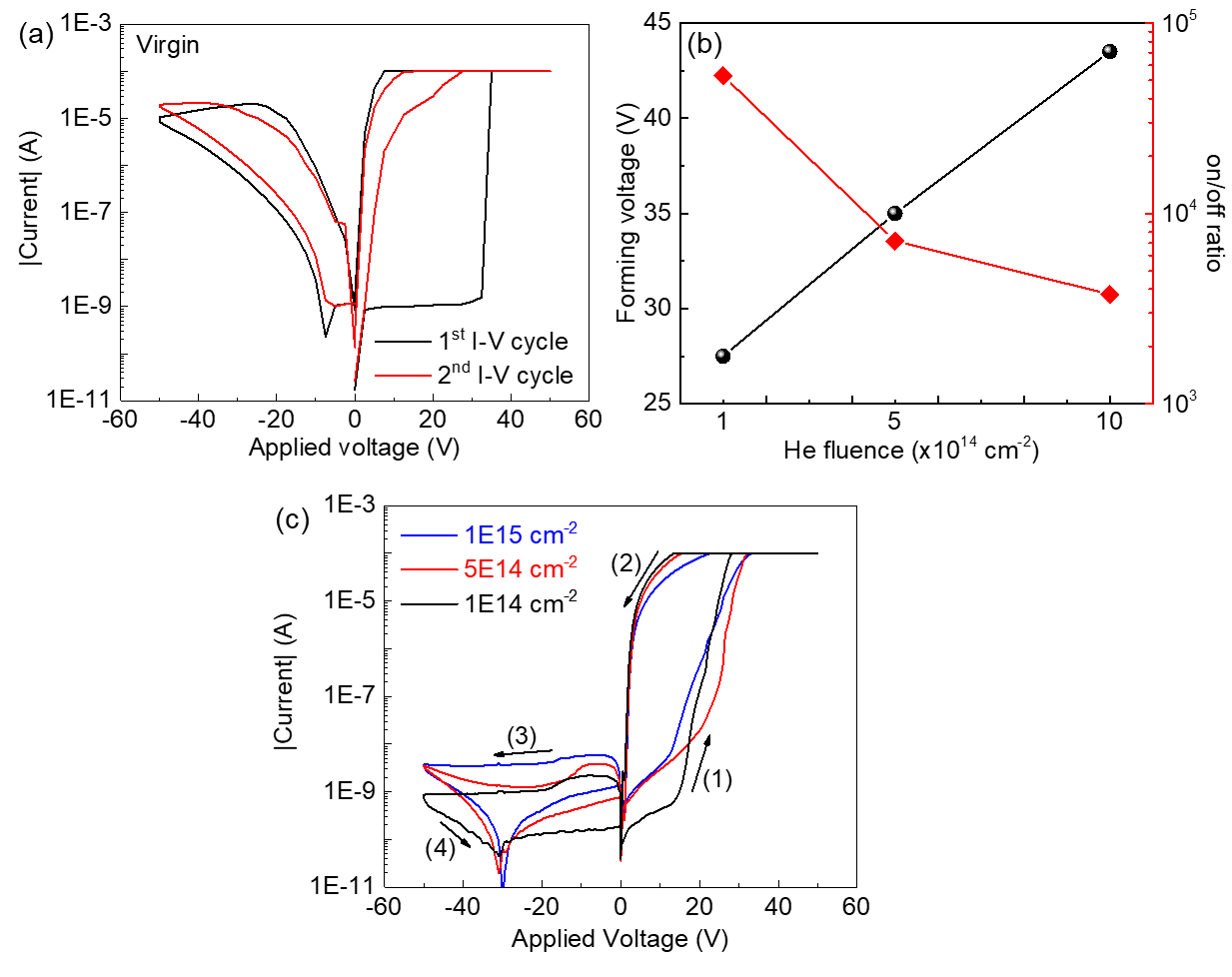


**Figure S2.** (a) I-V curves of the virgin sample. (b) Forming voltage and on/off ratio as a function of He implantation dose. (c) I-V curves of He-implanted samples with different He implantation dose.

Figure S2 (a) shows the I-V curves of the virgin samples. The virgin sample was broken down when the applied bias was ramped up to around +33 V for the first time and there is no significant current hysteresis observed in the second I-V cycle. Figure S2 (b) shows the forming voltage and on/off ratio as a function of He implantation dose. The electro-forming voltage increases but the on/off ratio with increasing He implantation dose. Figure S2(c) shows the I-V curves of the He-implanted samples with different He implantation dose. It is obvious that larger current in HRS and in negative bias range is obtained with increasing He implantation dose, which indicates that the conductivity of LNO thin films is increased by He implantation. However, the current in LRS shows the opposite pattern, which decreases with increasing implantation dose.


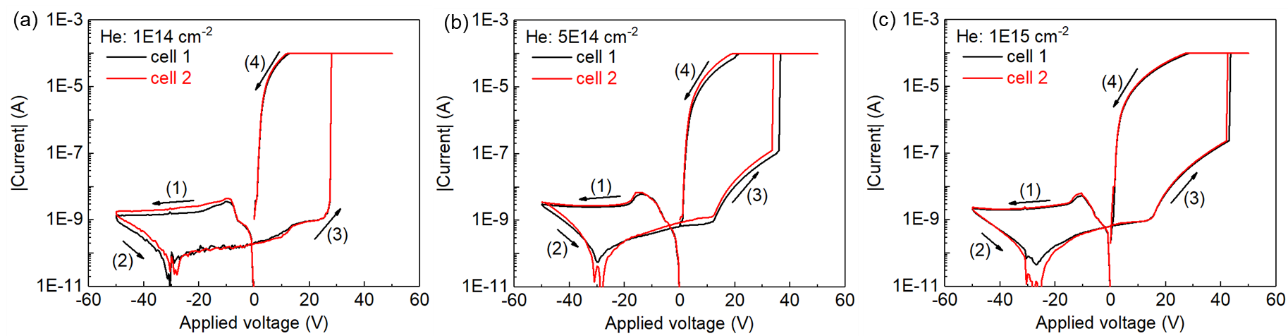


**Figure S3.** Electroforming process of 2 cells with the bias sweeping sequence of 0 V→-50 V→+50 V→0 V collected from the samples with He implantation dose of (a) 1E14 cm^-2^, (b) 5E14 cm^-2^ and (c) 1E15 cm^-2^.

Figure S3 shows the electroforming process with the bias sweeping sequence of 0 V→-50 V→+50 V→0 V collected from 2 cells of each He implanted sample. The I-V curves do not show significant different from that with bias sweeping sequence of 0 V→+50 V→-50 V→0 V as shown in Figure 2 (a)-(c).


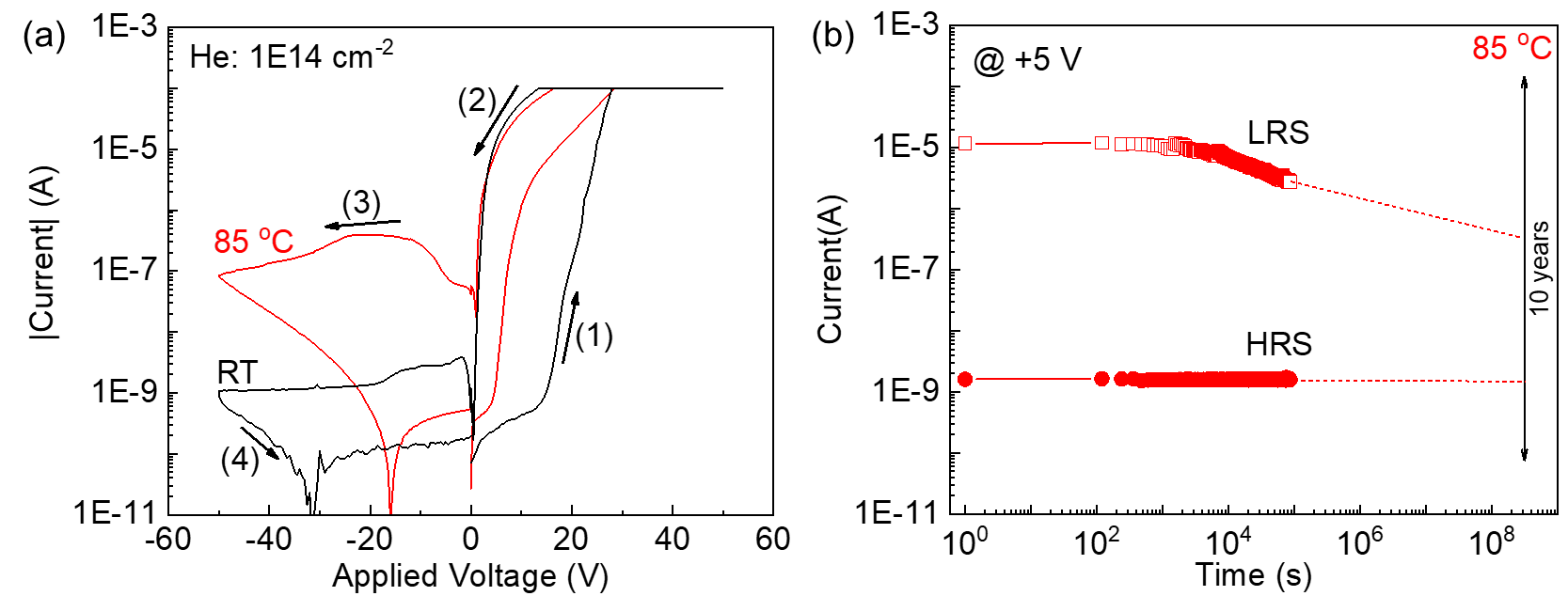


**Figure S4.** (a) I-V curves of the sample with He implantation fluence of 1E14 cm^-2^ at room temperature (RT) and 85 °C. (b) Retention test of the sample with He implantation fluence of 1E14 cm^-2^ at 85 °C for more than 24 hours. The extrapolated 10-year HRS/LRS retention time can be expressed by the dashed lines.

Figure S4 (a) shows the I-V curve of sample with He fluence of 1E14 cm^-2^ at RT and 85 °C. Even though the current of branch (1) in the I-V curve was increased significantly at 85 °C, the current hysteresis still exists in the positive bias range. Figure S4 (b) shows the retention test of sample with He fluence of 1E14 cm^-2^ at 85 °C. As similar to that at RT, the HRS is stable without significant variation during the testing period of 24 hours while the LRS initially shows an increasing resistance but then becomes stable after about 20 hours. The on/off ratio of the sample with He implantation fluence of 1E14 cm^-2^ at 85 °C is still above three orders of magnitude after 24 hours and above two orders of magnitude after 10 years as indicated by the dashed lines, which suggest stable resistive switching characteristics.

**Figure S5.** Typical I-V curves with compliance current of 1E-3 A for resistive switching devices on the sample with He implantation fluence of 1E14 cm^-2^ which were permanent broken.

The resistive switching was not stable with large compliance current. Figure S5 shows the typical I-V curves for the resistive switching devices on the sample with He implantation fluence of 1E14 cm^-2^ which were permanent broken (the red curve in Figure S5) after several resistive switching cycles with the compliance current of 1E-3 A.

**Table S1**. Comparison of the switching performance of LNO devices reported in this manuscript and some of the resistive switching devices reported in recent two years.

| Structure | E_set_/E_reset_ (V/cm) | on/off ratio | Retention time | Endurance  (cycles) | Switching speed | Reading power consumption for LRS/HRS (µJ) | Reference |
| --- | --- | --- | --- | --- | --- | --- | --- |
| GaIn/TiO_2_-CuO/ITO | 5.5×10^4^/  8.5×10^4^ | 3000 | 10^8^ s | 30 | - | - | 1 |
| TiN/Hf/HfO_x_/TiN | 5×10^7^/  5×10^7^ | 10000 | 10000 s | 5×10^7^ | 500 µs | 2×10^-6^/  2×10^-7^ | 2 |
| Ta/Ta_2_O_5_/Pt | 1.67×10^5^/  3.33×10^5^ | 10 | 10000 s | 10^12^ | 100 ms | 4×10^-4^/  4×10^-5^ | 3 |
| Pt/Ta_2_O_5-x_/TaO_2-x_/Pt | 1.25×10^6^/  8.75×10^5^ | ~10^3^ | - | >100 | 500 ns | 1.3×10^-5^/  7.5×10^-9^ | 4 |
| Ag/IGZO/MnO/Pt | 8.5×10^4^/  1.63×10^5^ | ~10^6^ | >5×10^4^ s @ 80 °C | >200 | - | - | 5 |
| TiW/HfO_2_/TiN | 3.2×10^6^/  2.6×10^6^ | 200 | 10^4^ s | 10000 | - | - | 6 |
| Au/LNO/Pt | 1×10^6^/  1×10^6^ | >3800 | >8×10^4^ s @ 85 °C | >5000 | 100 ms | 5/  2×10^-4^ | This work |

A comparison of the resistive switching performance of LNO thin films reported in this work and some of the resistive switching devices reported in recent two years is summarized in Table S1. The switching threshold electric fields (E_set_/E_reset_) of LNO devices is the average level among this table. By decreasing the thickness of LNO thin films, the operation voltage of LNO devices can be reduced, which needs to be further studied. The on/off ratio and retention time of LNO devices are comparable with those of the other recent reported resistive switching devices. The endurance of LNO devices was measured up to 5000 cycles in this work, which requires to be investigated in detail for further practical application purpose. The switching speed and the reading power consumption for LRS/HRS of LNO devices are tentatively much larger than those of the other recent reported resistive switching devices, which need to be further optimized. It is noted that the reading power consumption (*D_reading_*) was calculated by *D_reading_=V∙I∙t_reading_*, where *V and I* are the reading voltage and the reading current, respectively, and *t_reading_* is the pulse width of the reading pulse.


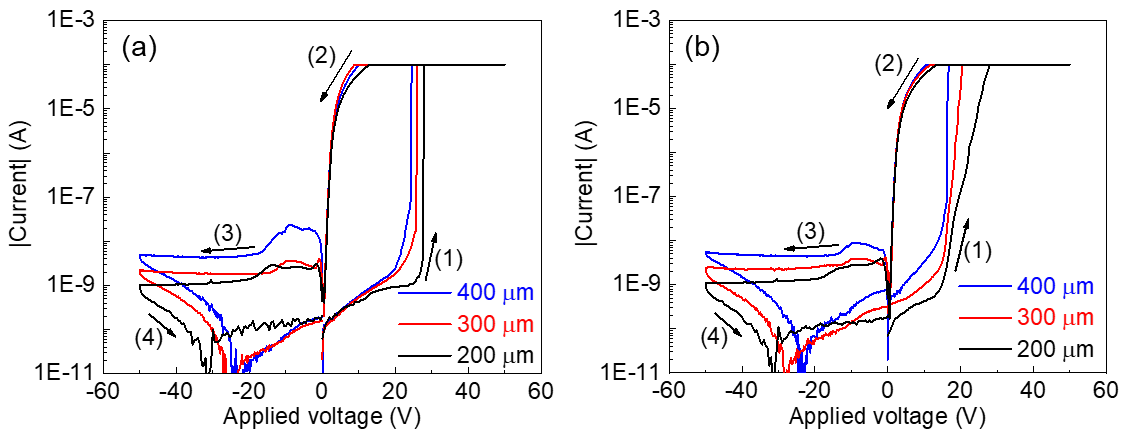


**Figure S6.** (a) Electroforming process and (b) I-V curves of the Au-LNO-Pt MIM structures (He implantation fluence of 1E14 cm-2) with cell diameter of 200 µm, 300 um, and 400 µm.

Figure S5 (a) shows the electroforming process of the samples with different cell diameters. By increasing the electrode diameter, the current is slightly increased before the electroforming, and the electroforming voltage is reduced. After the electroforming, the current in both HRS and negative bias range is increased obviously by increasing the electrode diameter, but LRS exhibit similar current levels as shown in Figure S5 (b). This suggests that the charge transport takes place over the entire area of the interface which is a sign of the interface-type resistive switching, while current in LRS is irrelevant to the electrode size which is a sign of the filamentary resistive switching.^7,8^ This also suggests the coexistence of the filamentary and interface resistive switching.

**References:**

1 Li, J.-C., Chen, B. & Qian, Y. J. C. A. P. Effect of fatigue fracture on the resistive switching of TiO_2_-CuO film/ITO flexible memory device. *Current Applied Physics* **18**, 953-960 (2018).

2 Su, Y.-T. *et al.* A method to reduce forming voltage without degrading device performance in hafnium oxide-based 1T1R resistive random access memory. *IEEE Journal of the Electron Devices Society* **6**, 341-345 (2018).

3 Zaffora, A. *et al.* Electrochemical tantalum oxide for resistive switching memories. *Advanced Materials* **29**, 1703357 (2017).

4 Hur, J.-H. & Kim, D.-k. J. S. r. A study on mechanism of resistance distribution characteristics of oxide-based resistive memory. *Scientific Reports* **9**, 302 (2019).

5 Abbas, H. *et al.* Reversible transition of volatile to non-volatile resistive switching and compliance current-dependent multistate switching in IGZO/MnO RRAM devices. *Applied Physics Letters* **114**, 093503 (2019).

6 Lin, C.-A., Huang, C.-J. & Tseng, T.-Y. J. A. P. L. Impact of barrier layer on HfO_2_-based conductive bridge random access memory. *Applied Physics Letters* **114**, 093105 (2019).

7 Sawa, A. Resistive switching in transition metal oxides. *Materials Today* **11**, 28-36 (2008).

8 Waser, R., Dittmann, R., Staikov, G. & Szot, K. Redox‐based resistive switching memories–nanoionic mechanisms, prospects, and challenges. *Advanced Materials* **21**, 2632-2663 (2009).
